# eEF2K Inhibitor Design: The Progression of Exemplary Structure-Based Drug Design

**DOI:** 10.3390/molecules28031095

**Published:** 2023-01-21

**Authors:** Kody A. Klupt, Zongchao Jia

**Affiliations:** Department of Biomedical and Molecular Sciences, Queen’s University, Kingston, ON K7L3N6, Canada

**Keywords:** eEF2K, protein translation, drug discovery, α-kinase, structure-based drug design, eEF2K inhibitor, A-484954, MHCKA

## Abstract

The α-kinase, eEF2K, phosphorylates the threonine 56 residue of eEF2 to inhibit global peptide elongation (protein translation). As a master regulator of protein synthesis, in combination with its unique atypical kinase active site, investigations into the targeting of eEF2K represents a case of intense structure-based drug design that includes the use of modern computational techniques. The role of eEF2K is incredibly diverse and has been scrutinized in several different diseases including cancer and neurological disorders—with numerous studies inhibiting eEF2K as a potential treatment option, as described in this paper. Using available crystal structures of related α-kinases, particularly MHCKA, we report how homology modeling has been used to improve inhibitor design and efficacy. This review presents an overview of eEF2K related drug discovery efforts predating from the 1990’s, to more recent in vivo studies in rat models. We also provide the reader with a basic introduction to several approaches and software programs used to undertake such drug discovery campaigns. With the recent exciting publication of an eEF2K crystal structure, we present our view regarding the future of eEF2K drug discovery.

## 1. Introduction

Structural biochemistry and biophysical analysis have been instrumental in the design and successful derivation of a vast number of current drugs on the market. Within the last several decades, these techniques have been supplemented with computational aids for drug discovery such as the ability to perform molecular dynamics or model proteins with incredible accuracy, recently demonstrated by AlphaFold [1,2]. Previously, our group reviewed a variety of strategies for structure-based drug design (SBDD) [3]. In this review, we present a specific example in which SBDD techniques have been profoundly impactful in developing such novel therapeutics.

Eukaryotic Elongation Factor 2 Kinase (eEF2K) is an atypical, calcium/calmodulin-dependent, α-kinase—part of a small kinase family with distinct domain structures from the hundreds of more promiscuous conventional protein kinases [4,5,6]. eEF2K phosphorylates the threonine 56 residue on eukaryotic Elongation Factor 2 (eEF2), a GTPase enzyme, responsible for aiding the ribosomal translocation of the elongating peptide between the aminoacyl and peptidyl ribosomal sites [7]. P-eEF2 (pT56) inhibits this translocation and peptide translation in the cell is halted as a result [7].

Though its α-kinase domain is closely related to other proteins in this respective family, the elaborate domain structure of eEF2K is distinct [8]. Closest to the N-terminus, between residues 75–100 is the unique calmodulin binding domain (CBD), conserved in eukaryotic homologs of eEF2K. The CBD contains a DXDXDG motif for Ca^2+^ binding and a 1-5-8-14 hydrophobic anchor binding motif for calmodulin (CaM) between phenylalanine 79 and methionine 95 [9,10,11]. The C-terminal domain (CTD) of eEF2K between serine 490 and glutamate 725 interacts with substrate eEF2 and is essential for kinase activity through substrate recognition [12].

As a master regulator of eukaryotic translational control, eEF2K is the downstream protein substrate of several signaling pathways and diseases, the focus of a few popular reviews [7,13,14]. eEF2K contains a regulatory loop (residues leucine 327 to serine 489) that links the α-kinase domain and CTD. It is worth a brief mention that the biochemical properties of eEF2K residue threonine 348, has been characterized quite extensively, and is implicated in a proposed two-step activation process of the α-kinase. In this hypothesized mechanism, the CaM binding of eEF2K enhances the affinity for the T348 to be phosphorylated by ~1000 fold. T348 is then rapidly phosphorylated and becomes associated with a basic residue pocket on the AKD [9]. An increase in pT348 has been associated with an increase in P-eEF2, evidence of an autoactivation mechanism [9].

The regulation of eEF2K is implicated in a wide variety of diseases that depart from normal cellular functioning [14]. Since eEF2 is solely inhibited through phosphorylation by eEF2K and eEF2K is an atypical α-kinase, the inhibition of eEF2K by small molecule inhibitors is a readily apparent target [14,15]. Compound this with the fact that eEF2K knock-out (KO) mice have been demonstrated to not display serious side effects and eEF2K KO is not required for fertility in male mice, eEF2K is presented as a unique drug target [14,16]. eEF2K has been deemed to be of importance as a target in cancer solid tumors, cardiovascular conditions, neurodegenerative diseases (including depression), and both hypoxic and nutritional stress [14,17,18,19,20,21,22,23].

Until recently, no structure of the entire eEF2K protein existed, yet despite this, several groups have been instrumental in characterizing the activity and properties of this unique enzyme [9,10,11,12,24,25,26]. This review will discuss protein modeling advances used to guide eEF2K inhibitor design, the effectiveness of a variety of eEF2K inhibitors, and the use of eEF2K inhibitors in disease models. Finally, we end this review with an overview of computational approaches to drug screening and a selected example of how researchers sought to combine an eEF2K inhibitor with a proteolysis-targeting chimera (PROTAC) warhead.

## 2. The Alpha Kinase Family

### 2.1. Overview of the Alpha Kinase Family

Initial work on the α-kinase family began in the mid 1990’s, in which a number of groups reported that myosin heavy chain kinase A (MHCKA) and eEF2K had no sequence homology to conventional protein kinases; yet later work by Yamaguchi et al. in 2001 revealed that despite the lack of sequence similarity, α-kinases have a similar catalytic core to those of conventional protein kinases [27,28]. Classification of these kinases as ‘α-kinases’ was established because the substrate phosphorylation sites of both MHCKA and eEF2K were alpha-helical [29]. More recent work on the α-kinase family however, has found α-kinase substrate phosphorylation on non-alpha helical regions, particularly the TRPM6 and TRPM7 channel-kinases [5,30].

To date, six α-kinases in humans have been identified and these include α-kinase 1 (ALPK1), α-kinase (ALPK2), α-kinase (ALPK3), TRPM6, TRPM7, and eEF2K [5]. ALPK2 and ALPK3 represent heart and muscle α-kinase, named after their respective tissues [29]. Closely related to eEF2K is MHCKA, from *Dictyostelium discoideum*, evidence of an ancestral relationship amongst α-kinases [31,32]. eEF2K is the only member of the α-kinase family that is calcium/calmodulin-dependent and contains a CaM binding domain adjacent to its catalytic domain. As opposed to other members of the α-kinase family, ALPK1 contains only an α-kinase domain. Other human α-kinases such as ALPK2/3 and TRPM6/7 contain Ig-binding domains and channel domains, respectively. For more information on α-kinase structure, substrate, and function, please see the comprehensive review by Middelbeek et al. [5].

### 2.2. Conventional Protein Kinase Inhibitors Do Not Inhibit Alpha Kinases

A distinguishable feature of the α-kinase subfamily is the unique ATP-binding active site compared to conventional protein kinases. Apart from structure differences, as observed in crystal structures, inhibitory experiments also provide evidence for this phenomenon. Initial work by Gschwendt et al. demonstrated eEF2K kinase activity to be inhibited effectively by rottelerin (IC_50_ of 5.3 μM), whereas eEF2K is resilient to staurosporin (IC_50_ > 50 μM), a non-selective kinase inhibitor that inhibits a vast number of conventional protein kinases [33]. Moreover, data from the lab of Alexey Ryazanov, revealed that use of staurosporin to inhibit ChaK1 (also referred to in the literature as TRMP7), another member of the α-kinase family, was unsuccessful at preventing kinase activity at concentrations up to 100 μM [4].

From a structural biology perspective, structural differences between that of α-kinases and conventional protein kinases provide strong evidence to explain the difference in kinase inhibition. Drennan and Ryazanov provided the field with an in-depth comparison between TRPM7 and PKA, a conventional protein kinase [4]. These differences include, but are not limited to, the presence of zinc fingers in α-kinases, a C-terminal lobe putative substrate-binding site, and a large α-helix in the N-terminal lobe of the α-kinase. Despite these differences only being characterized initially in 2004, future structural characterization would prove these to hold true for other α-kinases, a strong example of conservation in sequence resulting in conserved structure.

Crystal structures of MHCKA in complex with AMP-PNP, a non-hydrolysable ATP derivative, and eEF2K in complex with CaM, the latter of which published recently at the time of this writing, supports earlier characterization between TRPM7 and PKA [34,35]. Both MHCKA and eEF2K contain Cys 2—His 2 zinc fingers that coordinate a zinc ion, and an elongated α-helix. Active site differences between the α-kinase and conventional protein kinase, TRPM7 and PKA, respectively, has been previously summarized [4]. Briefly, in PKA, lysine residue 72, corresponding to lysine residue 1646 in TRPM7, forms a salt-bridge with glutamate 91. This glutamate residue approaches lysine 72 near the ATP ligand in the active site. However, in TRPM7, the corresponding glutamate residue 1718 approaches lysine from the opposite direction and forms hydrogen bonds with both lysine and adenine in ATP, furthermore, affecting charge in the pocket. Small differences such as those involved in forming active site salt-bridges help to explain a difference in inhibitory effectiveness. Moreover, differences in the hydrophobic pocket such as a phenylalanine or tyrosine residue (phenylalanine 1725) in TRPM7 form pi-pi stacking interactions with the adenine ring of ATP, and a catalytic loop without a lysine unlike conventional protein kinases may also contribute to differences in inhibitor binding.

### 2.3. The ‘Early’ eEF2K Inhibitors

In the 1990’s and early 2000’s, several key findings regarding eEF2K biochemical properties and the addition of resolving homologous structures permitted early research into eEF2K inhibition. We performed a number of searches using the ChEMBL database and BindingDB, both free online tools for compound searching based on target (in our case, human eEF2K) [36,37]. ChEMBL and BindingDB are maintained by the European Molecular Biology Laboratory and Skaggs School of Pharmacy and Pharmaceutical Sciences at the University of California, San Diego, respectively. Despite this, a PubMed.gov search, using tools provided by the NIH National Library of Medicine revealed literature with several other compounds not identified by databases, particularly from over two-decades ago. We attribute this to poor cataloguing and a lack of a collective effort at the time to catalogue protein inhibitors on the internet, unlike such efforts now in the structural biology community.

Without structural information, early work into eEF2K inhibition was primarily approached from a cellular biology angle. One such example was in 1996, in which Redpath et al. determined that insulin may result in decreased eEF2 phosphorylation in CHO cells [38]. This was also an important finding as it implicated eEF2K alongside metabolic conditions in the cell. Moreover, the same paper identified rapamycin, an immunosuppressant agent also resulted in a reduction in eEF2 phosphorylation [38].

**Figure 1:**
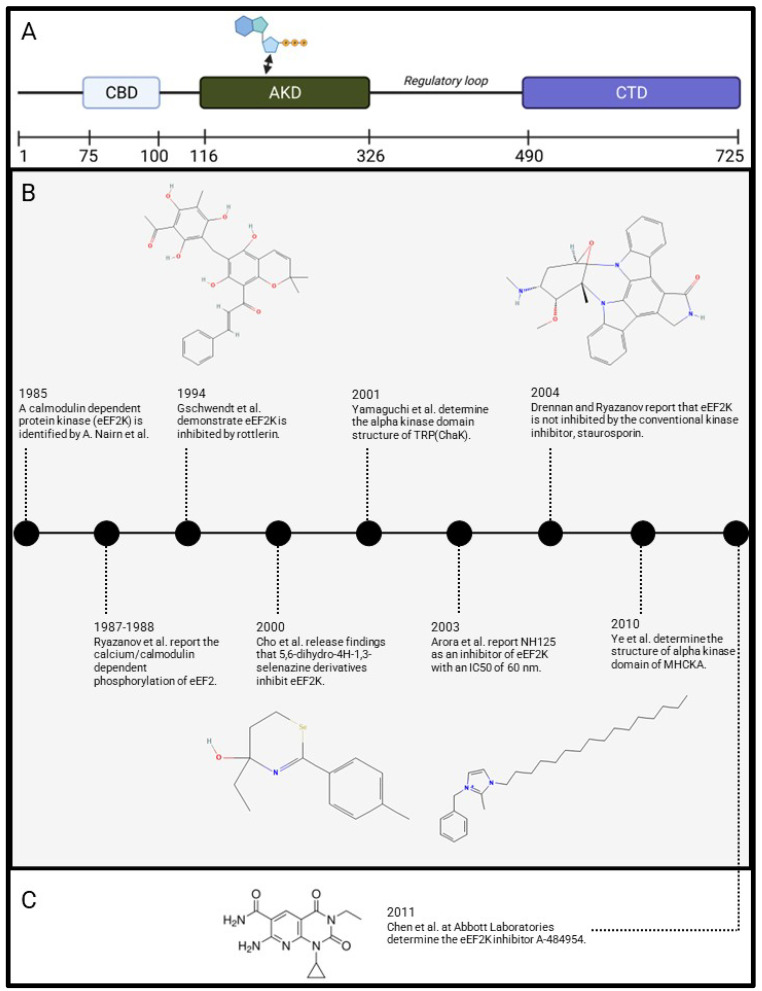
Milestones of eEF2K inhibitor development and 2D chemical structure of early eEF2K inhibitors. (**A**) Domain map of eEF2K including the calmodulin binding domain (CBD), alpha kinase domain (AKD), regulatory loop, and C-terminal domain (CTD). (**B**) Timeline begins with the 1985 identification of a ‘calmodulin-dependent protein kinase’ and subsequent development of ‘early’ eEF2K inhibitors. The additional publication of crystal structures of related α-kinases would greatly aid this drug discovery effort. (**C**) Structure of eEF2K inhibitor A-484954, a relatively recent inhibitor. Inhibitor structures obtained from PubChem database.

The earliest eEF2K inhibitor study, to the best of our knowledge, was reported by Gschwendt et al. in 1994. They demonstrated the inhibitory effect of rottlerin to reduce eEF2 phosphorylation with an IC_50_ of 5.3 µM (Figure 1) [33]. It was not far after that, in 2000, Cho et al. released findings, the earliest to our knowledge of a targeted eEF2K inhibitor. These 5,6-dihydro-4H-1,3-selenazine derivatives provided inhibition of eEF2K in a protein kinase assay in NIH3T3 cells [39]. Compounds ‘TS-2’ and ‘TS-4’ demonstrated enhanced inhibitory effects with an IC_50_ of 0.36 and 0.31 µM, respectively, a greater than 10-fold increase in inhibition, compared to rottlerin (Figure 1). This is quite remarkable given the limited structural information known about eEF2K at the time of this study. Three years later, in 2003, another drastic reduction in IC_50_ again was reported, this time through screening of imidazolium histidine kinase inhibitors. Compound NH125 was able to inhibit eEF2K kinase activity remarkably, with in vitro IC_50_ of 60 nM, in addition to glioma cell line experiments where NH125 treatment provided 10-fold resistance when eEF2K was overexpressed (Figure 1). Later work however, would suggest NH125 may however be responsible for induction of eEF2 phosphorylation as opposed to inhibition, an example of a potentially erroneous discovery process [40,41].

## 3. Structural Characterization of MHCKA Advanced eEF2K Drug Discovery

### 3.1. The MHCKA and eEF2K α-Kinase Domain Structures Demonstrate Conservation

It remains common in structure-based drug design for scientists to use homology modeling for acquiring predicted protein structure. The use of this technique has been well explained in several key reviews [42,43]. Briefly, with recent advances in computational power and programs such as AlphaFold, accurate homology modeling in drug discovery has become an adequate starting point for the selective filtering of inhibitor compound libraries [2]. Prior to the recent 2022 release of an eEF2K structure with its catalytic domain present, inhibitor design was entirely dependent on homology models.

Yamaguchi et al. resolved by X-ray diffraction the first α-kinase domain structure (prior to today’s naming conventions, was only referred to as an atypical protein kinase), from the TRP calcium channel (TRPM7) in *Mus musculus* (PDB: 1IAJ) [28]. This structure was also resolved in complex with ADP and AMP-PNP (PDB: 1IAH and 1IA9, respectively), providing useful structural insights. However, since percent identity between TRPM7 and other α-kinases is low (20.31% compared to MHCKA and 27.12% to eEF2K obtained using a UniProt alignment function [28,44]), it was unclear whether the structural knowledge would be “transferable” to other α-kinases.

**Figure 2:**
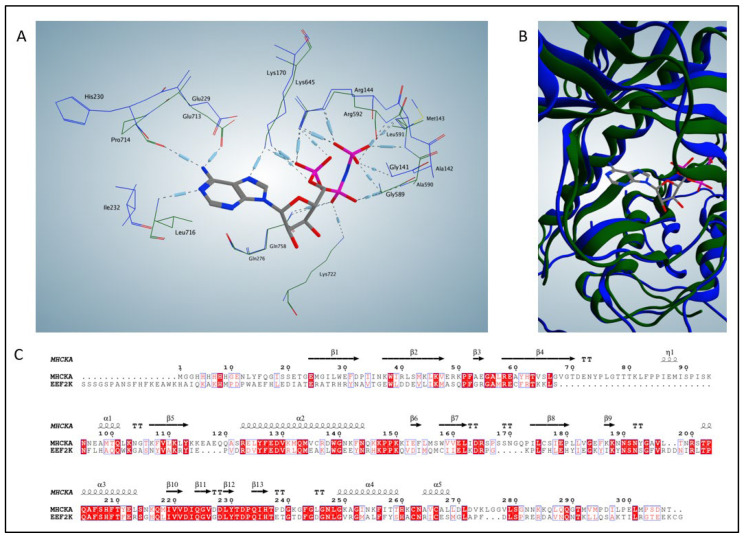
Alignment of MHCKA and eEF2K α-kinase domains demonstrates conserved structure and sequence. (**A**) Schematic view of the aligned active site residues of MHCKA (green) with bound AMP-PNP and eEF2K (blue), PDB; 5E9E and 7SHQ, respectively. (**B**) MHCKA (green) and eEF2K (blue) catalytic domains align to approximately an RMSD of 3.4 Å. (**C**) Sequence alignment of the catalytic domains of MHCKA and eEF2K with secondary structural elements of MHCKA displayed (top). Amino acid numbering beginning from the first methionine in the MHCKA 5E9E crystal structure. Figures prepared using Molecular Operating Environment 2022 and alignment prepared using ESPript version 3.0.

Our group determined the crystal structure of the α-kinase domain of MHCKA in complex with ADP, AMP-PCP, AMP, and ATP with a D766A (PDBs: 3LMH, 3LLA, 3LKM, 3LMI) [34]. Subsequently, our group performed more structural characterization of MHCKA [45,46,47]. The highest resolution observed in the collection of MHCKA structures was 1.6 Å, providing conformational details of the structural elements such as side chain positioning. We aligned the recent eEF2K crystal structure’s (PDB: 7SHQ) catalytic domain with the catalytic domain of MHCKA in complex with AMP-PNP (PDB: 5E9E) to demonstrate structural conservation (Figure 2). Root mean square deviation (RMSD), an averaged measurement of atomic distance, was approximately 3.4 Å between MHCKA and eEF2K. For reference, an RMSD of ~1 Å would indicate essentially identical structures.

Though it is worth noting that the crystal structure of eEF2K does not have observable electron density for a ligand in its active site and therefore, side chains may be more flexible [35]. Homologous residues between eEF2K (blue) and MHCKA (green) are highly conserved within the binding pocket (Figure 2) [35]. Important MHCKA residues for adenosine nucleotide binding such as E713, Q758, and K645 are conserved in three-dimensional space in the eEF2K crystal structure compared to MHCKA, indicative of a similar binding pocket and likely similar mechanisms. R592, in MHCKA, which is responsible for coordinating phosphate groups is also conserved in the eEF2K structure. Moreover, when comparing global structures of the catalytic domain, we observed structural similarity in secondary structural elements and tertiary domain (Figure 2B). This remarkable similarity in the active sites between α-kinases has increased confidence in the discovery process for eEF2K inhibitors, using only existing MHCKA structures.

### 3.2. Derivation of eEF2K Inhibitors from MHCKA Modeling

To assess the effect of MHCKA crystal structure models on eEF2K inhibitor design, we examined selected structure-based drug design literature citing the use of MHCKA PDB models [34,45,46,47]. One such example of this is the paper by Devkota et al. [48], which studied the compound ‘DFTD’ or 2,6-diamino-4-(2-fluorophenyl)-4H-thiopyran-3,5-dicarbonitrile, as an eEF2K inhibitor, and using kinetics and molecular docking with an MHCKA homolog model, identified the mechanism of inhibition—that is an irreversible nitrile group binds near the active site cysteine residue of eEF2K [48]. Other similar studies that utilized protein homology modeling with MHCKA include a study that probed the binding mechanism of A-484954, a popular eEF2K inhibitor [49]. In this effort, researchers performed in silico docking and subsequent molecular dynamics experiments to rank and identify compound binding conformation. It was predicted that inhibitors with larger cyclic groups (i.e., cyclopentyl opposed to cyclopropyl) may bind more favorably to a region in the eEF2K active site.

Such modeling efforts with the crystal structure of MHCKA to predict the eEF2K catalytic domain protein structure was also used more recently to evaluate benzamide tryptamine derivatives as inhibitors [50]. This study used the approach of predicting druggability in silico prior to using an MTT assay to monitor proliferative effects, revealing a relationship between activity and placement of the indole or benzene ring, respectively [50].

## 4. Inhibition of eEF2K with A-484954—A Promising Therapeutic

### 4.1. Development and Properties of A-484954

As briefly mentioned, work published after the discovery of eEF2K inhibitor NH125 (1-Benzyl-3-cetyl-2-methylimidazolium iodide) suggested that this once widely accepted inhibitor involved in eEF2K studies was inducing the phosphorylation of eEF2–counter to its intended purpose of inhibition [41]. Chen et al. explain that the perceived anti-cancer activity of NH125 may be correlated with eEF2 phosphorylation leading to inhibition of growth [41]. The authors of this same paper presented the selective eEF2K inhibitor, A-484954.

It is reported that A-484954 has an IC_50_ value of 0.28 μM against eEF2K and does not demonstrate substantial activity against an array of other kinases [41]. In a cancer cell line, A-484954 does not alter total eEF2 protein level but inhibits eEF2 phosphorylation [41]. A-484954 is commercially available from suppliers such as Sigma-Aldrich and MedChemExpress. It has been the subject of much investigation, including in animal models.

### 4.2. A-484954 Treatment in Animal Models Suggests Therapeutic Potential

Much of the in vivo work using eEF2K inhibitor A-484954 has been conducted by Tomoko Kodama and Hideyuki Yamawaki at Kitasato University, Japan. These in vivo studies in the literature have been conducted primarily in rat models, including spontaneously hypertensive rats (SHR), the first of which was by Usui et al., four years after the initial paper characterizing A-484954 [51]. Increased eEF2K expression was identified in the mesenteric artery (i.e., the artery that provides oxygenated blood and nutrients to the digestive system including the intestines) of SHRs [51]. Using A-484954 at a dose of 10 μM, the researchers determined that this eEF2K inhibition inhibited platelet derived growth factor induced smooth muscle cell proliferation [51]. Moreover, it also restricted platelet derived growth factor induced smooth muscle cell migration [51]. Using the CaM inhibitor (W-7 at 50 μM), platelet derived growth factor induced phosphorylation of eEF2K was also inhibited [51]. This research was able to establish the role that eEF2K has in mediating platelet derived growth factor induced smooth muscle cells by activating several signaling pathways, suggesting that it may serve as a potential therapeutic target for diseases related to hypertension.

Shortly after this initial study, Kodama et al. published findings regarding how A-484954 affects the contraction properties of blood vessels isolated from rat aorta and the mesenteric artery [52]. It was demonstrated in this study that A-484954, thus inhibition of eEF2K, induces relaxation of the mesenteric artery via opening of the smooth muscle Kir (potassium channel) and endothelium derived nitric oxide [52]. This relaxation property was inhibited by a Kir blocker [52]. More recent work the same group would reveal that in SHRs, A-484954 treatment resulted in lower blood pressure at 15 weeks of age, and furthermore, mesenteric artery relaxation at up to 9 weeks of age [53]. It has been demonstrated that this vasorelaxation effect occurs when eEF2K is inhibited, thus promoting the production of endothelium-derived relaxing factors [53]. This group has also demonstrated similar findings [54,55,56,57,58]. Due to eEF2K’s implications in a variety of disease pathways, A-484954 serves as an exciting tool to alter elongation control in a variety of unhealthy states as has been demonstrated in cardiovascular sciences in vivo and cancer studies in vitro [14]. To our knowledge, thus far there have been no eEF2K inhibitor clinical human trials.

### 4.3. An A-484954 PROTAC—A Case of Innovative eEF2K Inhibition

Targeted inhibition of enzymes can often be an exceptionally difficult endeavor. Proteolysis-targeting chimeras, or PROTACs, provide the capability to recruit an E3 ubiquitin ligase near a protein of choice (target protein) to promote protein degradation by native cellular mechanisms [59]. Those interested may also find the referenced database, ‘PROTAC-DB’, useful for exploring PROTAC design [60]. Recently, Liu et al. published findings of an eEF2K targeting PROTAC [15]. It would be useful to describe the methodology and findings in this innovative case of drug design.

In brief, protein ligand, in this case A-484954 (commonly referred to as a warhead), is connected by a small molecule linker to an E3 ligand. This promotes eEF2K to be in proximity to E3 ligase. E3 ligase recognizes E2 ligase to promote the ubiquitination of nearby eEF2K, followed by subsequent eEF2K degradation by the proteasome. To aid in their study, Liu et al. used MHCKA crystal structure as a template for protein homology modeling and subsequent docking of A-484954 into the ATP binding pocket [15]. From this docking, a linker attachment site was identified extending outwards from the binding pocket and PROTACs were designed with several different linker designs [15]. A-484954 was also modified with either a cyclopropyl or ethyl moiety [15]. It was observed that in breast cancer carcinoma cells, the lead PROTAC reduced total eEF2K protein levels and phosphorylation of eEF2, and importantly apoptosis was observed in this cancer model when treated with PROTAC [15].

## 5. Inhibition of eEF2K as a Method of Disease Treatment

### 5.1. Neurological Disorders

The role of eEF2K has been implicated in various neurological disorders including Alzheimer’s disease (AD) and depression [14,17,18,61]. eEF2K as a calcium/calmodulin-dependent protein kinase that controls global translation, plays a role in synaptic plasticity through regulating protein synthesis, particularly for long term memory, and is regulated by Ca^2+^—notably an important secondary messenger in human neuron cells [20]. The importance of eEF2K in brain functioning was demonstrated in eEF2K mutated (loss of function) mice, that displayed a phenotype of a defective associative taste learning and abnormal brain function activation, visualized by magnetic resonance imaging [19]. In AD, an accumulation of amyloid-β plagues, which impedes brain functioning, through impaired plasticity and potentiation, results in the activation of AMP-activated protein kinase (AMPK) [61]. eEF2K may be an inhibitory target for restoration of the AD phenotype as elevated levels of phosphorylated eEF2 are present in the hippocampus of AD model mice [19].

It is hypothesized that by transiently inhibiting elongation, the expression of proteins encoded by weak mRNA that compete poorly for ribosomal translation will be translated, and some of these proteins may contribute to restoring proper homeostasis as eEF2K is transiently activated and inhibited [14,62]. This idea is particularly important in a study demonstrating the actions of ketamine on eEF2K. Ketamine treatment of major depressive disorder blocks synaptic NMDA receptors, which can deactivate the calcium-calmodulin activation mechanism of eEF2K [18]. A decrease of eEF2 phosphorylation by ketamine administration allows the synthesis of brain-derived neurotrophic factor protein and subsequent antidepressant response [18].

### 5.2. Cancer

One may expect the uncontrolled proliferation of cancer cells is tied to a dysregulation in protein synthesis and suppression of elongation by phosphorylated eEF2, mediated by eEF2K, is needed to prevent tumor growth. This contrasts with work published in several different tumor types including breast and lung cancers. Cancer cells rely heavily on glycolysis, also referred to as the ‘Warburg effect’, which is required for their expansion [63]. Work by Cheng et al. determined, however, that eEF2K promotes glycolysis in cancer cells [64]. eEF2K deficiency reduces glucose uptake and the production of both lactate and ATP in Ras-transformed mouse embryonic fibroblasts [64]. Since eEF2K may influence tumor cell glycolysis, inhibition of eEF2K in cancer remains a worthwhile avenue for future exploratory studies.

Such an example of eEF2Ks detrimental effects in cancer is its expression in lung cancer being associated with poor patient survival [65]. It was also determined that eEF2K is upregulated significantly in lung cancer cell lines and inhibition by siRNA or rottlerin results in the suppression of lung cancer proliferation and survival [65]. This eEF2K silencing by siRNA also resulted in the significant inhibition of lung cancer tumors in mice [65]. siRNA targeting eEF2K has also been demonstrated to decrease eEF2 phosphorylation and inhibit tumor growth in mice with triple negative breast cancer [66].

## 6. Computational Aids for eEF2K and General Drug Discovery

**Figure 3:**
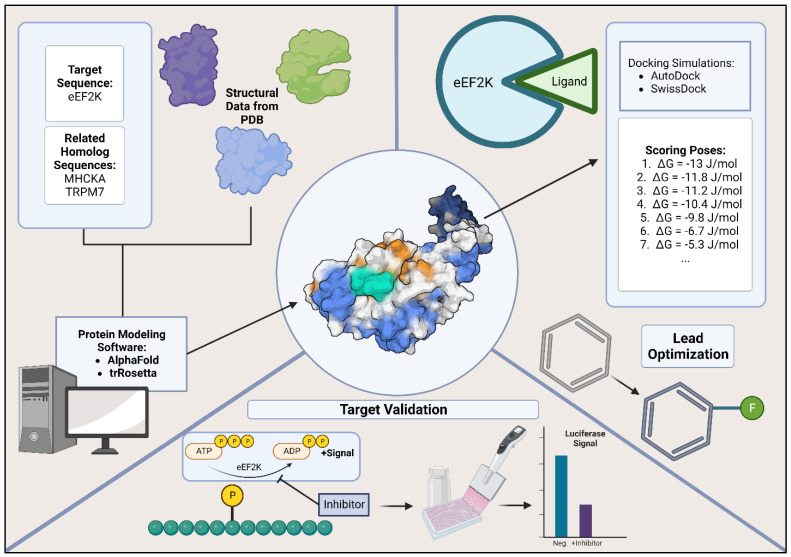
Simplified eEF2K inhibitor structure-based drug design pathway. The structure-based drug design process includes protein structure determination or accurate protein model development. Protein-ligand interactions may be screened with docking simulations and leads are optimized. In the case of eEF2K, a simple target validation may be executed to monitor the conversion of ATP to ADP and Pi using a luciferase assay.

Several computational tools can be used, most of them free for academic use, for research groups interested in developing inhibitors that may target eEF2K, or other proteins in general. Though not encompassing, we describe a general workflow for structure-based drug design and the optimization process (Figure 3). A recent example of drug discovery targeting eEF2K, which used a computational screening pipeline with a database of traditional Chinese medicines, identified several antitumor chemicals with low cytotoxicity [67].

### 6.1. Accurate Protein Modeling is Essential to Drug Discovery and Development

Fundamental to structural biology is the idea that the primary sequence of a peptide has a direct impact on the tertiary structure of proteins. This simplistic view does not explain the effects that protein environment, chaperones, post-translational modifications, and other biophysical effects, have on a proteins three-dimensional structure leading to the challenge of accurate protein design and homology modeling. X-ray crystallography, NMR (nuclear magnetic resonance spectroscopy), and cryo-EM (cryogenic electron microscopy) experiments, seek to produce the three-dimensional structures of proteins at sub nanometer resolution. Having an accurate protein structure provided by these experimental methods has been crucial to drug discovery when building a ligand that targets a protein with specificity. However, the question arises—should a proteins structure not be available in the widely used and accessible Protein Data Bank (PDB), and what is the best way to predict and model the protein target [68]?

This problem is so core to structural biology, that it has even prompted competitions such as the ‘Critical Assessment of Techniques for Protein Structure Prediction’. Most recently, CASP14, which highlighted the accuracy of the program AlphaFold2 when modeling monomeric proteins [2,69]. AlphaFold2 has become a preferred program of choice for protein modeling, since it is freely available to use and edit, has a database available online for quick retrieval of protein structure, and provides predicted confidence scores at the residue level [2,70]. AlphaFold2 uses biophysical restraints, a powerful multi-sequence alignment, and the recent development of machine learning to predict protein structure [2]. Other machine learning programs that predict protein structure notably include the trRosettta server [71]. Developing a highly accurate starting model for drug discovery will affect downstream operations such as ligand screening, therefore, we recommend using a variety of programs to compare structure prediction outputs.

### 6.2. Protein-Ligand Docking Simulations and Free Energy Score

Succeeding accurate protein modeling is identifying potential lead compounds using docking. Small-molecule inhibitors may be obtained from databases such as ZINC in which their 3D coordinates can be imported into various docking programs [72]. Other options not discussed further in this paper include generating an inhibitor by small-molecule modifications or fragment-based design in which a target binding pocket is ‘filled’ with functional groups of an inhibitor, eventually being linked by a scaffold or backbone [73]. These rather advanced techniques are however available in the literature for those interested. Protein-ligand docking programs vary substantially in price, though many basic programs are available online as servers free for academic use [74,75].

Prior to protein-ligand docking simulation, one should consider the target binding site in their protein. An example of this is the ATP binding site of eEF2K. Binding sites may be learned from literature (e.g., the AdoMet site of a methyltransferase) or by prediction, where a program, such as ‘PrankWeb’, will identify pockets on a protein surface [76]. After a binding site and library of ligands are selected, the docking process is relatively simple as most programs automate this process. One example is AutoDOCK, a free program that ranks protein-ligand docked poses by free energy (i.e., ΔG), where the assumption is made that a lower free energy indicates a more favorable conformation between protein and ligand [77]. It is worth noting that despite a protein-ligand conformation being energetically favorable its output should still be visually inspected. For example, if developing a binding site inhibitor for eEF2K that is similar in structure to ATP, one would expect a similar binding mode with a low RMSD between the inhibitor and ATP.

### 6.3. Lead Compound Modification and In Vitro Evaluation

In the event of a successful protein-ligand docking campaign, several lead inhibitor molecules will be obtained with reasonable binding modes. Several programs allow researchers to go a step further and optimize their compounds prior to synthesis and testing. This process, optimization, involves making minor changes to the lead inhibitors to confer ideal properties such as but not limited to, reduced toxicity, improved specificity, solubility, etc. One such program is the Molecular Operating Environment (MOE). One general workflow for this is to first predict pharmacophores (key functional features) of lead inhibitors. This identification allows one to computationally edit molecules for ideal properties while maintaining core binding capability. Other considerations for improving protein-ligand binding are to computationally modify lead inhibitors and re-score them post-modification. By making small modifications randomly on the various terminal groups of the inhibitor the number of lead molecules will expand and can be re-scored for free energy value after optimization.

After synthesis, but prior to expanded in vitro testing and in vivo trials, lead compounds should ideally be screened in simple assay with a direct readout that indicates inhibition of the protein target. In the case of eEF2K, Xiao et al. have published work for such a high-throughput assay [78]. Interestingly, this assay uses a peptide substrate for phosphorylation by eEF2K that is designed for MHCKA phosphorylation due to reports that the eEF2 threonine 56 recognition peptide is not a suitable substrate for this assay [78]. In this assay, eEF2K, CaM, ATP, and peptide substrate, are all plated by liquid handler robot. Inhibitor compounds are also added and after the reaction is completed, the reaction is stopped with ADP-Glo reagent for luminescence [79]. This assay is suitable for lead compound selection because it provides a quantifiable readout that can be compared statistically to that of known inhibitors, does not involve radiolabeled reagents like other kinase assays, and the readout is directly proportional to enzymatic activity.

## 7. Finally a Crystal Structure: What Does It Mean for eEF2K Drug Design in the Future?

Excitingly, in 2022, Piserchio et al. deposited a CaM bound eEF2K crystal structure with phosphorylated threonine 348 (PDB:7SHQ) with an accompanying publication [35]. This undoubtedly will play a significant impact in future eEF2K targeted drug design. To date, to the best of our knowledge, no eEF2K inhibitors reported so far have been designed using crystal structure as opposed to a homology model. While the recent structure does not have ATP or a derivative bound in its active site, the deposited crystal structure captures the activated eEF2K enzyme with CaM bound and the autophosphorylation of threonine 348 in its respective pocket [35]. With existing protein-ligand docking programs, and ATP derivative—MHCKA crystal structures serving as binding pose templates, it is not unreasonable to suggest that the ATP-eEF2K binding pose may be accurately predicted. Moreover, following the release of an eEF2K crystal structure it may be worth investigating the effect that small molecule inhibitors can have when binding to the CaM binding pocket on eEF2K to disrupt the activation mechanism, which would give rise to higher selectively than targeting the ATP binding site.

Estimates from the early 2000’s suggest that determination of an X-ray crystal structure may provide value in the amount of up to USD 20 million and the ability to filter lead compounds more accurately [80]. X-ray structures provide the ability for scientists involved in drug design to build pharmacophores, key parts of a molecule involved in biological interactions, with improved accuracy and purpose [80]. More importantly, the potential for new inhibitor design offered by crystal structure determination for eEF2K may encourage the development of valuable new therapeutics that regulate peptide elongation in various diseases.

## 8. Concluding Remarks

Over the span of several decades, tremendous efforts have been made to characterize eEF2K and develop inhibitors that target this important atypical kinase. While earlier inhibitors developed without the use of homology models or computational techniques can successfully inhibit eEF2K mediated eEF2 phosphorylation, more recent inhibitors such as the modified PROTAC linked A-484954 have demonstrated great therapeutic potential. The deposition of related α-kinase crystal structures in the PDB has provided several research groups with the ability to design novel, or modify existing eEF2K inhibitors, using a variety of software tools, most free for academic use. It remains to be demonstrated the effects of eEF2K inhibitors in human clinical trials, a critical step in structure-based drug design campaigns.

## Data Availability

No new data was generated for this review.
